# Draft Genome Sequences of the Vibrio parahaemolyticus Strains VHT1 and VHT2, Pasteurization-Resistant Isolates from Environmental Seafood

**DOI:** 10.1128/mra.00793-22

**Published:** 2022-10-17

**Authors:** Guadalupe Meza, Hussain Majrshi, Kumar Saurabh Singh, Ratnakar Deole, Hung King Tiong

**Affiliations:** a Department of Biological and Environmental Sciences, University of West Alabama, Livingston, Alabama, USA; b Clinical Laboratory, Huntsville Hospital, Huntsville, Alabama, USA; c Bioinformatics Group, Wageningen University, Wageningen, The Netherlands; d Oklahoma State University, Center for Health Sciences, Department of Biochemistry and Microbiology, Tulsa, Oklahoma, USA; Montana State University

## Abstract

Two pasteurization-resistant strains, VHT1 and VHT2, of environmental, viable but nonculturable, pathogenic Vibrio parahaemolyticus were isolated from environmental oysters. Their whole-genome sequences were constructed. The genome sizes for VHT1 and VHT2 are 5.11 Mbp and 5.26 Mbp, respectively.

## ANNOUNCEMENT

Vibrio parahaemolyticus is an opportunistic, viable but nonculturable (VBNC), seafood-borne pathogenic bacterium (1) that has been closely monitored by the CDC’s Cholera and Other *Vibrio* Illness Surveillance (COVIS) system since 1989 and that is frequently difficult to detect in the food system (2). In the present study, two VBNC, pasteurization-resistant strains of environmental V. parahaemolyticus, VHT1 and VHT2 (3), were analyzed and their whole-genome sequences determined. Here, we present the draft genome sequences of V. parahaemolyticus VHT1 and VHT2.

Previous work by Meza et al. (3) used a modified two-step enrichment technique to recover VHT1 and VHT2 from environmental oysters. Briefly, *Vibrio* contaminants in a 25-g sample were homogenized in 225 g saline peptone water (SPW) in a stomacher bag with a Stomacher 400 Circulator lab blender and incubated for 48 h for enrichment at 35°C. Subsequently, the enrichment solution was heated at 80°C for 20 min and refrigerated overnight, and culture dilutions made in sterile buffered peptone water (BPW) were plated onto thiosulfate-citrate-bile salts-sucrose agar medium, followed by incubation for 72 h at 35°C. Visible green colonies of presumptive V. parahaemolyticus were then streaked onto Brain Heart Infusion (BHI) agar supplemented with 3% NaCl for purification, and the pure cultures were resuspended in BHI storage solution before being stored at −70°C. Bacterial 16S sequences (400 to 600 nucleotides) were identified using the NCBI nucleotide BLAST (blastn) platform. The sequences matched those of V. parahaemolyticus strain DHO76 (GenBank accession no. CP066246.1) perfectly.

Using the Qiagen DNeasy blood and tissue kit, the VHT1 and VHT2 genomes were extracted from the fresh cultures prepared in BHI broth supplemented with 3% NaCl, followed by overnight incubation at 35°C and a repeated subculture (3). The DNA extracts were analyzed using the Quant-iT PicoGreen double-stranded DNA (dsDNA) assay kit coupled with the Qubit fluorometric quantifier and the NanoDrop spectrophotometer (DNA quantity and purity measurement). Paired-end libraries with 2,930,163 (VHT1) or 2,627,568 (VHT2) reads were generated using the Nextera XT DNA sample preparation kit, followed by whole-genome sequencing (WGS) using the Illumina MiSeq platform (2 × 250 bp, with the MiSeq reagent Nano kit v2) at Molecular Cloning Laboratories. FastQC genome reads with quality (Phred score) values of >20 (4) were used for subsequent processing with Trimmomatic v. 0.32 (5). Genome assemblies were constructed using SPAdes v. 3.13.0 (6) and evaluated using the basic metrics listed in Table 1. Annotations of genome assemblies and putative pathways were carried out using the NCBI Prokaryotic Genome Annotation Pipeline (PGAP) v. 4.12 (7) and KEGG tools (8), respectively, and are listed in Table 1. Default parameters were used for all software unless otherwise specified.

**TABLE 1:** Assembly and annotation metrics of V. parahaemolyticus VHT1 and VHT2

Statistic	Data for strain:	
	VHT1	VHT2
No. of quality-filtered paired-end reads	2,930,163	2,627,568
No. of scaffolds	57	271
*N*_50_ (bp)	807,591	531,181
Largest scaffold length (bp)	1,194,203	1,737,051
Assembly length (bp)	5,115,539	5,261,907
Avg genome coverage (×)	181.3	112.5
G+C content (%)	45.24	45.12
No. of genes	4,769	5,141
No. of protein-coding genes	4,586	4,909
No. of rRNA genes	18	17
No. of tRNA genes	114	119
No. of pseudogenes	47	92
No. of genes associated with KEGG pathways	1,009	985
No. of genes associated with KEGG orthology	2,457	2,415
GenBank accession no.	JACERE000000000	JACERD000000000
SRA accession no.	SRR12395870	SRR12395869
BioProject accession no.	PRJNA608845	PRJNA608845

The sequencing data statistics are tabulated in Table 1. The genome sizes of both VHT1 and VHT2 are within the range of those for V. parahaemolyticus variant strains, namely, between 4.29 and 6.47 Mbp (NCBI; accessed 10 July 2021), and they have G+C contents of 45.24% (VHT1) and 45.12% (VHT2).

### Data availability.

This paired-end whole-genome MiSeq project has been deposited at GenBank under the BioProject accession no. PRJNA608845 (VHT1 and VHT2). The reads have been deposited in the SRA under the accession no. SRR12395870 (VHT1) and SRR12395869 (VHT2). The 16S rRNA gene sequences were deposited at the NCBI under the GenBank accession no. OP090156 (VHT1) and OP106961 (VHT2). Bacterial culture material, genomic, and identification details are available at https://doi.org/10.6084/m9.figshare.20457063.
